# Pertussis in Early Infancy: Diagnostic Challenges, Disease Burden, and Public Health Implications Amidst the 2024 Resurgence, with Emphasis on Maternal Vaccination Strategies

**DOI:** 10.3390/vaccines13030276

**Published:** 2025-03-05

**Authors:** Konstantina Leontari, Alexandra Lianou, Andreas G. Tsantes, Filippos Filippatos, Zoi Iliodromiti, Theodora Boutsikou, Styliani Paliatsou, Anastasios E. Chaldoupis, Petros Ioannou, Alexandra Mpakosi, Nicoletta Iacovidou, Rozeta Sokou

**Affiliations:** 1 Neonatal Department, School of Medicine, Aretaieio Hospital, National and Kapodistrian University of Athens, 11528 Athens, Greece; kleontari@yahoo.gr (K.L.); stpaliatsiou@yahoo.gr (S.P.); niciac58@gmail.com (N.I.); 2 Neonatal Intensive Care Unit, General Hospital of Nikaia “Agios Panteleimon”, 18454 Piraeus, Greece; 3 Microbiology Department, “Saint Savvas” Oncology Hospital, 11522 Athens, Greece; 4 First Department of Pediatrics, National and Kapodistrian University of Athens, ‘Aghia Sophia’ Children’s Hospital, 11527 Athens, Greece; 5 Laboratory of Haematology and Blood Bank Unit, School of Medicine, “Attiko” Hospital, National and Kapodistrian University of Athens, 12462 Athens, Greece; achaldou@med.uoa.gr; 6 School of Medicine, University of Crete, 71003 Heraklion, Greece; 7 Department of Microbiology, General Hospital of Nikaia “Agios Panteleimon”, 18454 Piraeus, Greece; alexiabakossi@yahoo.gr

**Keywords:** *Bordetella pertussis*, pregnancy, vaccination, outbreak, public health

## Abstract

*Bordetella pertussis* is the causative agent of pertussis or whooping cough, an acute and highly contagious respiratory infection that can have serious and fatal complications such as pneumonia, encephalopathy, and seizures, especially for newborns. The disease is endemic not only in the European Union (EU)/European Economic Area (EEA) but also globally. Larger outbreaks are anticipated every three to five years, even in countries where vaccination rates are high. Despite the high pertussis vaccination coverage in developed countries and a low rate of pertussis incidence for many years, especially during the COVID-19 pandemic, the incidence of pertussis has been on the rise again, with outbreaks in some places, which is referred to as “re-emergence of pertussis”. The aim of this review is to underscore the critical importance of achieving high vaccination coverage, particularly among pregnant women, to safeguard vulnerable neonates from pertussis during their early months, before they are eligible for vaccination. This aligns with the need to address diagnostic challenges, mitigate disease severity, and strengthen public health strategies in light of the ongoing 2024 *Bordetella pertussis* resurgence.

## 1. Introduction

*Bordetella pertussis* is a Gram-negative (−), aerobic, pathogenic encapsulated coccobacillus bacterium of the genus of Bordetella and the causative agent of pertussis or whooping cough, an acute and highly contagious respiratory infection [1]. All age groups are affected by the disease, though newborns and infants under three months of age are most at risk for severe complications [2] such as pneumonia, apnea, leukocytosis, pulmonary hypertension, hypoxemia, heart failure, encephalitis, seizures and encephalopathy and even death [1,3]. *B. pertussis* is spread from person to person by direct contact with respiratory secretions or by aerosolized respiratory droplets. The mortality rate for pertussis in neonates and infants up to two months old is 1–2% [4].

Up to 90% of household contacts (people in the family environment who are not immune) and 50% to 80% of schoolroom contacts will become infected after exposure [5]. Newborns, especially those under three months old, are at significant risk for severe disease from pertussis due to their immature immune systems and inability to be fully vaccinated. In recent years, especially following the disruptions caused by the COVID-19 pandemic, many European countries such as Austria, Croatia, and Denmark have seen an increase in pertussis cases among infants, with a concerning rise in hospitalizations [6,7].

Recently Italy has been experiencing a significant pertussis outbreak, particularly affecting newborns and young infants. The outbreak primarily affects a high proportion of newborns and young infants who are either unvaccinated or incompletely vaccinated [8]. In contrast, Denmark’s epidemic has shown the highest incidence among adolescents [9]. This difference can likely be attributed to varying rates of vaccine coverage during pregnancy; while approximately 85% of pregnant women in Denmark are vaccinated, vaccination coverage among pregnant women in Italy remains unknown due to the absence of a national registry for prenatal vaccinations [8]. The introduction of maternal vaccination has been crucial for protecting infants, yet many pregnant women remain unvaccinated. In Italy, up to 94% of mothers were found to be unvaccinated, and 80% reported receiving no information about prenatal vaccination. The high incidence of pertussis in newborns can be linked to these gaps in maternal vaccination coverage. Furthermore, during the current Italian outbreaks, three deaths have been reported in newborns who were too young to begin their vaccination schedules, underscoring the urgent need for targeted vaccination campaigns to protect this vulnerable population. Additionally, a significant percentage of infants had accessed healthcare before the onset of the disease, suggesting that timely recognition of pertussis could have potentially prevented secondary cases. This emphasizes the importance of screening for respiratory symptoms and providing post-exposure prophylaxis to high-risk contacts [8].

Given the high transmissibility of pertussis, returning travelers could potentially introduce additional cases upon their return to their home country. Considering the broad geographic range of exposure, healthcare providers should maintain a high index of suspicion for pertussis in travelers arriving from temperate and tropical regions, as well as from developed and developing countries [10]. International travel has increased by 50% over the past decade, with 983 million tourist arrivals recorded in 2011 [11]. The rise in long-distance travel, particularly to emerging economies in Asia and Africa, is significant. Travel frequency is also increasing among individuals with comorbidities, professionals traveling for work, and those visiting friends and relatives [12]. Travelers visiting friends and relatives, defined as migrants, as well as their spouses or descendants traveling to their country or region of origin, constitute a high-risk group for travel-related illnesses [13]. Health professionals should advise individuals seeking pre-travel information or those who fall ill post-travel and must be aware of the variations in the likelihood of specific known and unknown diseases, depending on the traveler’s characteristics and destination, including pertussis [14,15]. The epidemiological situation, along with gaps in immunization, combined with the continuous increase in global travel and mass gatherings, may contribute to the heightened spread and transmission of pertussis worldwide. Pre-travel consultations offer an opportunity to assess immunization status and administer routine vaccines, including the pertussis vaccine.

The impact of pertussis vaccines on mortality is a key indicator for the World Health Organization, and trends in mortality rates and age distribution can inform strategies for vaccinating pregnant women. In a systematic review [16], data from 19 eligible studies reporting pertussis mortality rates (PMR) per million population were analyzed. During a prevaccination observation period (≥50 years) in high-income countries (HICs), PMRs decreased by >80% in both infants and children aged 1–4 years, in parallel with improvements in living conditions. In low- and middle-income countries (LMICs), PMRs prior to the introduction of vaccination were comparable to the higher rates recorded in HICs during the prevaccination period. After the introduction of vaccination in HICs, there was a further significant reduction in deaths (>98%), but at the same time, a significant shift towards younger ages among the remaining deaths was observed [16,17,18,19,20]. In LMICs, the limited available data also indicate significant and rapid reductions in PMR, initially in older infants and children [21,22,23]. However, long-term data are lacking to fully capture the distribution of remaining deaths. In Sweden, a significant increase in the prevalence of undetectable antibodies to pertussis was recorded in the decade following the implementation of the mass vaccination program for children with the acellular vaccine [24]. Corresponding data are not available from LMICs using the whole-cell vaccine in the basic vaccination program without booster doses. Recording residual infant deaths and the prevalence of maternal seropositivity could provide valuable information for planning pertussis vaccination during pregnancy in LMIC settings, especially if more precise immunological correlates of infant protection against pertussis-related death were available.

This review aims to outline epidemiological data, diagnostic challenges, disease severity, and public health implications associated with the 2024 resurgence of *Bordetella pertussis*, with a particular focus on maternal vaccination strategies. By emphasizing the critical importance of achieving high vaccination coverage—especially among pregnant women—this review seeks to highlight the need to protect vulnerable neonates from pertussis in their early, pre-vaccination months. This work also aims to contribute to the development of effective public health strategies by addressing current diagnostic limitations and evaluating disease impact.

## 2. Clinical Features

The classic pertussis disease is characterized by moderate upper respiratory tract symptoms that usually appear 7–10 days (range: 5–21 days) after exposure. During the first one to two weeks following the onset of a pertussis infection, known as the catarrhal stage, symptoms are non-specific and may include coryza (inflammation of the mucous membranes), low-grade fever, and a mild cough [5,25]. In infants, the cough may be minor, or apnea may be the predominant symptom [5]. During the following weeks (paroxysmal stage), the cough intensifies, characterized by episodes (paroxysms) of coughing, occasionally leading to vomiting after coughing [25]. Typically, the distinct sound known as the “whoop” can be heard in children and teenagers who have not been exposed to the infection before, during episodes of sudden and intense coughing. The frequency of coughing paroxysms varies, and they are often followed by vomiting. Fever is absent or minimal in most cases. The coughing paroxysms eventually subside into a softer, less frequent cough, but during the convalescent stage, paroxysms may flare up again in response to further respiratory infections. The clinical case definition of pertussis includes cough with paroxysms lasting more than two weeks, whooping, post-tussive vomiting, or apnea with or without cyanosis.

In immunocompromised individuals, including infants, adolescents, and adults, the catarrhal stage of the illness may be quite moderate. This is then followed by a prolonged cough, which can last anywhere from two weeks to several months, with or without sudden and uncontrollable fits of coughing [5,25].

Gagging, gasping, apnea, and a brief catarrhal stage are some of the early symptoms of atypical disease that can affect infants under six months of age. In infants under two months old, the case–fatality ratio is approximately 1–2%. When children, adolescents, and adults have received vaccinations in the past, the sickness may be less severe and they may not experience the typical whooping and paroxysmal cough [1].

Studies have already indicated that as much as 13% of prolonged coughing is caused by pertussis [26,27,28]. Individuals are extremely contagious from the acute phase of the disease until the early intense stage (within the first two weeks), with their ability to spread the infection gradually decreasing over the following three weeks [12]. Patients who receive the correct antibiotic treatment within 21 days after the start of the disease are no longer contagious after five days of treatment [5,25]. Due to pertussis’ vague presentation in its initial stage, which is also extremely contagious, there may be a delay in confirmation of pertussis diagnosis. Transmission may already occur, particularly to those in close proximity, during this period [5].

## 3. Diagnosis and Treatment

Diagnosing and preventing the transmission of pertussis can be hard due to the variation in clinical presentation based on age and immunization threshold [25].

Centers for Disease Control and Prevention (CDC) guidelines for laboratory confirmation of pertussis include culture and PCR when the above clinical case definition is met. Serology is not included as a confirmatory test in the current case definition for reporting purposes. Testing for *B. pertussis* is widely available in commercial laboratories [27].

The sensitivity, specificity, and interpretation of the diagnostic tests for *B. pertussis* that are currently available can be impacted by various factors, including the illness stage, antibiotic use, prior vaccination status, specimen collection and transport conditions, and the utilization of non-standardized tests. White blood cell (WBC) count significantly increases (20–50 × 10^9^/L) during the paroxysmal phase, with lymphocytes predominating. However, this is less pronounced in vaccinated individuals, making leukocytosis an unreliable diagnostic marker. C-reactive protein (CRP) levels generally remain within normal limits, while blood chemistry should be monitored in hospitalized patients to assess hepatic and renal function. The fluorescent quantitative polymerase chain reaction (qPCR) method is commonly used for detecting *Bordetella pertussis* nucleic acids in respiratory samples, whereas next-generation sequencing (NGS) has identified *B. pertussis* DNA in blood, from patients with severe disease, who were classified as cases of bacteremia or bloodstream infection due to the severity of their clinical condition in these reports [29,30]. However, this definition has not been definitively established nor adequately verified. Serological testing, particularly the detection of PT-IgG antibodies, is useful for retrospective diagnosis, especially in cases persisting for more than one month, but its reliability may be influenced by recent vaccination [31]. When the aforementioned clinical case criteria are satisfied, the Centers for Disease Control and Prevention (CDC) recommends using PCR and culture for laboratory confirmation of pertussis.

The prevalence data presented in a systematic review by Muloiwa et al. [32] suggest that LMICs (low- and middle-income countries) may also be experiencing a resurgence of pertussis, similar to what is observed in high-income countries (HICs). The Global Vaccine Alliance (GPI) and the World Health Organization (WHO) advocate for strengthening surveillance systems as a critical component of pertussis control. However, pertussis surveillance in LMICs remains insufficient, leading to gaps in the accurate epidemiological data observed in this study. The review highlights that the method used for laboratory confirmation of cases impacts the quantification of the pertussis disease burden. Notably, PCR should be prioritized for pertussis confirmation in LMICs, as its higher sensitivity is more likely to accurately capture the true burden of disease and provide a clearer understanding of the global epidemiological pattern of pertussis across different settings. In contrast, culture, which is considered the “gold standard” of diagnosis, missed an average of 85% of cases detected by PCR in studies that used both methods [32].

Physicians can use a macrolide antibiotic (azithromycin, clarithromycin, or erythromycin) to treat pertussis in patients older than one month; azithromycin is the recommended medication for neonates(<1 month). Transmission to others can be limited by antimicrobial medication therapy with a macrolide antibiotic given <3 weeks after the onset of the cough [1]. Severe pertussis requires intensive care alongside antibiotic therapy, with key interventions including mechanical ventilation for respiratory failure, therapeutic plasma exchange for hyperleukocytosis, and targeted pulmonary vasodilator therapy for pulmonary hypertension [33,34,35]. Inhaled nitric oxide (iNO) may be used for pulmonary hypertension, although its efficacy in pertussis remains uncertain [33,35]. Early initiation of extracorporeal membrane oxygenation (ECMO) is recommended for critically ill patients with severe pulmonary hypertension, where hemodynamic collapse may occur rapidly [36]. Additionally, intravenous pertussis immunoglobulin (P-IVIG) may be administered as adjunctive therapy [31].

In addition, the effective treatment of pertussis, especially in severe cases, requires adequate healthcare infrastructure, including access to antibiotics and supportive care. In LMICs, these resources may be limited, leading to higher rates of complications and mortality, particularly among infants and other vulnerable populations [37]. Without appropriate treatment and vaccination programs, pertussis can spread rapidly, not only within local communities but also to neighboring countries or across borders, exacerbating the global burden of the disease. In regions where environmental and social factors hinder access to healthcare, it is crucial to develop and implement alternative strategies for the control and prevention of pertussis. Addressing these challenges is essential not only for the local prevention and treatment of pertussis but also for preventing global outbreaks and safeguarding public health worldwide.

## 4. Prevention

Nowadays, pertussis remains a public health problem of great concern, with high rates of morbidity and mortality. WHO estimates that about 24.1 million cases of pertussis and 160,700 children under 5 years of age die of pertussis in a year [37]. According to the United States Centers for Disease Control and Prevention, about one-third of infants with pertussis require hospitalization, and 1–2% of those cases die [25].

The immunity conferred by childhood vaccination and natural disease wanes with time; therefore, adolescents and adults who have not received a tetanus–diphtheria–pertussis (Tdap) booster vaccination can become infected or reinfected with pertussis. Infants, especially those too young to be protected by a complete vaccination series, are at the highest risk for severe illness and death from pertussis.

Pertussis is a preventable disease owing to vaccination, and the introduction of pertussis immunization has led to a decrease in the number of pertussis cases and deaths among children since the establishment of the WHO Expanded Programme on Immunization in 1974 [38].

### 4.1. Bordetella Vaccines

It is well known that *B. pertussis* cannot survive for long periods without a human host, as it is entirely dependent on it. Initially, *Bordetella* was considered an extracellular pathogen, leading researchers to assume that antibodies and humoral immunity were the most effective forms of protection. This assumption was based on the ability of antibodies to neutralize extracellular agents through opsonization, neutralization, and complement activation [39,40]. However, both *B. pertussis* and other *B. species* such as *B. bronchiseptica* have been shown to survive intracellularly in various cell types, such as macrophages [21,41,42]. Therefore, humoral immunity alone may not be sufficient to induce a long-term immune response. Indeed, studies in children and animal models have demonstrated that T cells are essential for protection against Bordetella [43,44]. Furthermore, large clinical trials have shown that anti-Bordetella antibody titters do not correlate perfectly with protection against *B. pertussis* [39]. To date, no clear immunological correlations of protection against *Bordetella species* have been identified. However, studies in humans, baboons, and other mammals suggest that effective protection requires a combination of humoral immunity and a Th1/Th17-type cellular response [43].

Managing pertussis presents several challenges. Following the introduction of immunization programs, the primary mode of transmission has shifted from child-to-child outbreaks to adult-to-child spread. As a result, infants and young children are now at a higher risk of infection [45,46]. Moreover, due to the significant side effects of the whole-cell pertussis vaccine, the acellular pertussis vaccine was introduced in the late twentieth century and has since been widely adopted in various countries and regions. While the acellular pertussis vaccine (aPV) causes fewer adverse effects compared to the whole-cell pertussis vaccine (wP) [47], numerous studies have indicated that aPV is linked to an increased risk of pertussis infection over time [48,49,50]. The acellular pertussis vaccine primarily protects against severe disease rather than preventing infection. As a result, individuals vaccinated with the acellular pertussis vaccine may still become asymptomatic or mildly symptomatic carriers, potentially contributing to the transmission of pertussis within the population [51]. Additionally, since the first case of erythromycin-resistant *Bordetella pertussis* was identified in a 2-month-old infant with pertussis in Arizona, USA, in 1994, subsequent reports of antibiotic-resistant *Bordetella pertussis* have emerged in several countries, including France, China, Iran, and Vietnam [52].

In recent years, the rise in pertussis cases has raised concerns about the level of protection provided by current vaccines. Both aPV and wP vaccines are in use, with aPV being primarily administered in developed countries, while wP remains the preferred choice in most developing nations. A comparison of the immune responses induced by these vaccines has shown that children vaccinated with either wP or aPV develop Th1 responses. However, the aPV elicits a mixed Th1/Th2 response [53]. In experimental models, both natural infection and the wP vaccine stimulate Th1 and Th17 responses, whereas the aPV predominantly induces Th2 and Th17 responses, with only a weak Th1 response [54]. Several studies suggest that wP vaccines provide better protection than aPV against both disease and bacterial colonization. For instance, in mouse models, wP vaccines have been shown to prevent colonization in both the lungs and nasal cavity following intranasal challenge, whereas the aPV only limits colonization in the lungs. A decrease in vaccine immunity over time has become a serious concern, and new pertussis vaccines are being evaluated to solve this problem [55].

The recent resurgence of pertussis in vaccinated populations highlights the limitations of current pertussis vaccination programs. Although parenterally administered pertussis vaccines provide a high level of protection, particularly against severe pertussis, they do not prevent nasal carriage or transmission of *B. pertussis* [51,56]. In fact, nasopharyngeal carriage of *B. pertussis* in hosts who have received aPV can be prolonged, thereby increasing the continued spread of the bacteria through transmission, which may have contributed significantly to the current resurgence of the disease. Reducing nasal carriage through immunization is important to lower the risk of exposure and reduce transmission, particularly in unvaccinated individuals. Prolonged immunity is also a critical goal for new pertussis vaccines, as the rapid waning of immunity is a major issue with current aPV [57]. Natural infection with *B. pertussis* has been shown to provide long-term protection against subsequent infections, although immunity induced by infection is typically not lifelong [58]. However, prolonged immunity through infection may reflect the induction of persistent mucosal immunological memory, which can be quickly reactivated in the respiratory mucosa upon rechallenge. *B. pertussis* infections induce strong local secretory and Th17-type cellular immune responses, which are protective against *B. pertussis* infection [59]. These types of immune responses are not effectively induced by the parenteral administration of current pertussis vaccines.

Current aPVs induce robust circulating IgG production and prevent severe disease in children, adults, and infants born to vaccinated mothers. However, these vaccines do not prevent nasal infection, allowing transmission of *B. pertussis* also through asymptomatic carriers [60]. Animal model studies have demonstrated that, unlike natural infection, immunization with aPV fails to induce the secretion of secretory immunoglobulin A (IgA) or interleukin-17 (IL-17) by tissue-resident memory CD4 T cells (TRM), which are critical for sustained sterilizing immunity in the nasal mucosa. Live attenuated or aPV with novel adjuvants that promote the production of respiratory IgA and TRM cells, particularly when administered nasally, are currently under development and hold significant promise as next-generation pertussis vaccines [60]. However, compared to the wealth of studies published on systemic pertussis vaccination, mucosal vaccination has attracted relatively little attention. Mucosal vaccination with aPV, combined with various adjuvants or nanoparticle formulations, has shown promising results in mice. However, these vaccines only induce immune responses to a limited number of antigens, which may lead to the emergence and spread of vaccine-evading variants, especially if their immune response is strong [61]. This is well illustrated by the increase in *B. pertussis* isolates with pertactin (PRN) deficiency after the administration of PRN-containing aPV [62]. The use of outer membrane vesicles (OMVs) with a broad antigen repertoire would be less prone to vaccine escape mutations but would likely require multiple administrations to induce strong local protective immunity [57,58,59,61]. A recent phase 2/3 clinical trial [63] provides compelling evidence that the new recombinant acellular pertussis vaccines (aPgen and TdaPgen) elicit a stronger and more sustained immune response compared to traditional chemically detoxified vaccines (Tdapchem). The study found that pertussis toxin (PT) antibody levels in adolescents who received Tdapchem returned to baseline within two years, whereas those vaccinated with aPgen or TdaPgen maintained significantly elevated antibody levels even after 2–3 years. These findings suggest that the new recombinant vaccines may offer longer-lasting immune protection, potentially improving the effectiveness of pertussis booster immunization strategies.

Given the identification of new *B. species* and their role in human infections, the potential for vaccines to protect against multiple *B. species* has garnered significant interest. It has been suggested that existing aPV, which contain a limited number of bacterial proteins, may be less effective than the wP vaccine against other *B. species* due to antigenic shifts and vaccine-induced adaptations [64]. With Bordetella infections on the rise in both humans and animals, there is an urgent need for improved vaccines that offer broader protection. Researchers are exploring several innovative vaccine strategies and delivery methods presented in Table 1.

Routine administration of acellular pertussis vaccines with diphtheria and tetanus toxoids (DTaP) is recommended in infancy at ages 2, 4, 6, and 15–18 months, and at 4–6 years. Booster doses are recommended at the age of 11, and during pregnancy. Pertussis immunity is not permanent, regardless of whether it is acquired through vaccination or infection. For the initial few years following vaccination, pertussis vaccines offer substantial protection against symptomatic infection, particularly severe cases [50]. Subsequently, vaccination induces a protective immunity that diminishes as time progresses [50,58]. This diminishing immunity effect is also observed following pertussis infection [58]. Vaccine-derived immunity diminishes within the first two to twelve years of vaccination [58,74,75], whereas infection-derived immunity diminishes within the first four to twenty years of infection [58]. Individuals who had only received acellular vaccines may experience a more rapid rate of decline than those who had received whole-cell vaccines (or priming with a whole-cell vaccine after receiving acellular vaccine doses) [50].

It is crucial to ensure that all the age cohort being vaccinated has a high level of vaccination coverage in order to protect them from infection. However, it is essential to recognize that the immunity of this cohort diminishes as it ages. In order to achieve effective pertussis control, the optimal timing of vaccination supplemental doses must be taken into account, taking into account the waning immunity of children and adolescents [58].

To ensure continued protection against tetanus and diphtheria, administer booster doses of either Td or Tdap every 10 years throughout an adult’s life. Immunization remains suboptimal, with coverage of the three-dose infant vaccine (DPT3) remaining low in LMICs overall, particularly in low-income countries [76]. Even in areas where DPT3 coverage is deemed acceptable, doses are often not administered on time, undermining the protective effect of the vaccine in young infants [77,78].

Unlike LMICs, where pertussis resurgence may be a result of inadequate administrative capacity, in HICs this may be linked to low and delayed coverage due to vaccine hesitancy. These issues have contributed to the resurgence of several diseases, including pertussis, largely driven by misinformation spread through the internet and social media. Vaccination hesitancy—a major factor associated with delayed or missed immunizations in infants and children—remains a key factor that increases the risk of pertussis infection in these age groups [79]. Vaccination hesitancy refers to a psychological state, vaccination behavior, or decision-making process [80]. Maternal vaccine hesitancy has been associated with lower vaccination coverage, including the pertussis vaccine (0–74%) [81], resulting in fewer infants acquiring maternal antibodies [82]. Additionally, parental hesitancy and non-medical exemptions have contributed to the increase in delayed or missed immunizations during childhood. Vaccination hesitancy is a complex and context-dependent phenomenon that fluctuates over time and across regions, influenced by factors such as complacency, convenience, and trust. It is observed in both developed and developing countries and is shaped by various determinants [83]. Increased access to the internet and social media has notably altered public attitudes toward vaccination, particularly during disease outbreaks [84]. While healthcare professionals remain the primary source of health information, many parents rely on the Internet for vaccine-related guidance. However, anti-vaccination content on social media can negatively impact public opinion and exacerbate vaccine hesitancy. Research has shown that exposure to anti-vaccine websites or blogs can influence attitudes and further increase hesitancy toward vaccination [85,86].

### 4.2. Vaccination During Pregnancy

Vaccination during pregnancy plays an essential role in antenatal care, safeguarding the health of the mother, the fetus, and the newborn against communicable diseases. Vaccination during pregnancy provides active immunity to the mother and passive immunity to the newborn, through the transfer of maternal antibodies both transplacentally and via breast milk [87,88]. Therefore, it protects three distinct populations: the pregnant woman, the developing fetus, and the newborn/infant. Evidence supporting that maternal vaccination with Tdap reduces the risk of pertussis in infants led to the rapid establishment of official national recommendations in the USA (2011) [88] and the UK (2012) [78]. In the following years, maternal vaccination was recommended in many EU/EEA countries, as well as Canada, Australia, and most countries in Central and South America [89,90]. However, several populous and developed countries, such as China and Japan [91,92], as well as EU/EEA countries like Estonia, Finland, Bulgaria, Malta, and Slovakia, have not yet implemented national Tdap vaccination programs for pregnant women [83].

**Figure 1:**
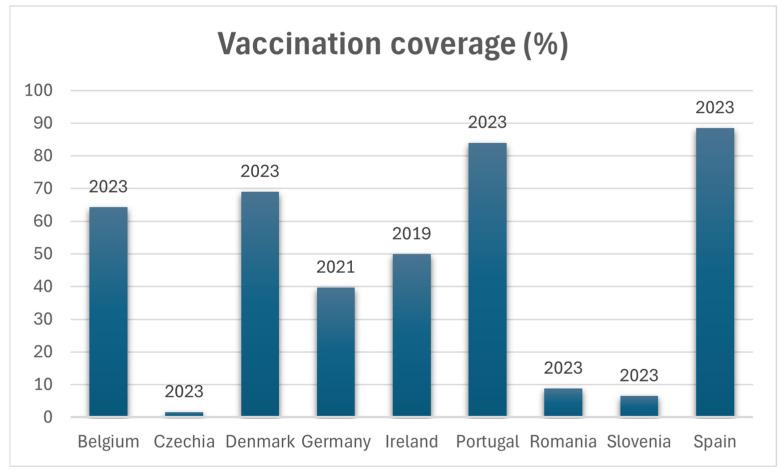
Percentage of vaccination coverage across countries of the EU/EEA.

Even in countries where maternal vaccination is actively promoted, coverage remains below the desired level. According to data from the commercial and multistate databases of Market Scan and Medicaid, in the USA in 2017, six years after the establishment of the Advisory Committee on Immunization Practices (ACIP) recommendation, maternal vaccination coverage was only 56.3% (Commercial) and 31.4% (Medicaid) [93]. These rates remained stable both before and after the COVID-19 pandemic [48], with coverage reaching 55.4% during the 2022–2023 influenza season [49]. In the EU/EEA, in 2023, only nine countries reported official data on maternal vaccination coverage, with significant variations, from just 1.6% in the Czech Republic to 88.5% in Spain (Figure 1) [83].

Furthermore, some countries reported a decrease in vaccination coverage in 2023 compared to previous years, suggesting reduced compliance with official recommendations. In England, from October 2012 to 3 September 2013, the average vaccination coverage of pregnant women was 64% [25]. Coverage peaked at 76% in December 2016, but then gradually declined, reaching 58% in June 2023 [85].

Concerns about the possibility that maternal vaccination could affect the effectiveness of primary vaccination of infants appear to be limited. Studies have shown that children born to vaccinated mothers exhibit a lower immune response to primary immunization against pertussis, with reduced antibody concentrations to multiple antigens of the pathogen [94,95], as well as lower antibody avidity [96]. Additionally, it has been shown that the immune response after maternal vaccination may be heterologous, negatively impacting not only the pertussis vaccine but also the immune response to other vaccines, such as polio and pneumococcal conjugate vaccines, which contain modified diphtheria or tetanus toxins as carrier proteins [97]. However, epidemiological assessments of the incidence of pertussis among children born to vaccinated mothers who received the recommended doses of the pertussis vaccine indicate that the risk is minimal and may be offset by natural fluctuations in the frequency of pertussis over time [98].

Two approaches are used to protect infants before their vaccination: vaccination of the mother during pregnancy and vaccination of all the infant’s close relatives (cocooning) [99]. Maternal immunization with a vaccine that contains acellular pertussis with reduced antigen can provide protection to infants who are not yet eligible for vaccination through passive maternal antibody transfer.

To protect infants, pertussis vaccination in pregnant women, recommended between 16 and 32 weeks of gestation for each pregnancy, was introduced to the CDC’s guidelines in 2010. The UK and Australia introduced this recommendation in 2012 and 2015, respectively [100].

In Switzerland, the National Immunization Technical Advisory Group (Eidgenössische Kommission für Impffragen, EKIF) recommended in 2013 that pregnant women receive a booster dose of the acellular pertussis vaccine, combined with tetanus and diphtheria toxoids (Tdap) if their last dose was administered more than five years prior [32].

In 2017, this recommendation was revised, and since then, Tdap has been advised during the second trimester (between the 13th and 26th week of gestation) for every pregnancy, regardless of the time elapsed since the last dose [33]. Regardless of prior vaccination, individuals should receive a Tdap dose during each pregnancy.

While Tdap can be administered at any point during pregnancy, the ideal timing for maximizing the maternal antibody response and transferring passive antibodies to the infant is between 27 and 36 weeks of gestation, with the recommendation to administer it during the earlier part of this time window [101].

A Swiss observational study revealed that a higher level of maternal antibody transmission was associated with earlier Tdap vaccination in pregnancy, possibly as a result of the longer transfer period before delivery [102]. The findings from the recent Optimising the Timing of Whooping Cough Immunisation in Mums (OpTIMUM) randomized clinical trial revealed that the highest levels of pertussis-specific IgG antibodies in umbilical cord serum were observed when mothers received the Tdap vaccine early in the third trimester (28–32 weeks’ gestation), in comparison to a vaccination before 24 weeks’ or between 24 and 27 weeks’ gestation [103].

Each booster dose will temporarily increase the amount of maternal anti-pertussis toxin antibodies which are transported transplacentally to the fetus, thereby providing protection for the newborn for at least 3 months [25]. The vaccination is safe and effective in preventing severe pertussis disease in neonates and infants [104].

Boosting in the second half of every pregnancy results in high levels of passive immunity in infants and is critical to the protection of the newborn [105]. Fewer infants will be hospitalized for and die from pertussis when Tdap is given during pregnancy rather than during the postpartum period. Maternal pertussis immunization leads to the passive and active transfer of maternal antibodies providing protection for the infant [106,107]. The pertussis vaccine is one of the three vaccines (influenza, pertussis, and COVID-19) that are strongly recommended during pregnancy in order that protection is provided to the neonate [108]. It takes about 2 weeks after Tdap receipt for the mother to have protection against pertussis. The Tdap vaccination among pregnant women has been found to reduce the risk of hospitalization in infants <2 months old [101]. All available safety data on Tdap during pregnancy are certainly reassuring [2]. The protective role of breastfeeding against pertussis in infants should not be overlooked. Breastfeeding provides passive immunity through maternal antibodies and potentially cellular immune factors, offering critical protection to newborns during the early months of life before they complete the primary vaccination series [109,110]. Data from animal studies suggest that both breastfeeding and in utero imprinting influence neonatal mucosal cellular immune responses to *B. pertussis* infection. The mechanism responsible for transferring cellular immunity from vaccinated mothers to their offspring through breastfeeding may involve the uptake of antigen-presenting cells (APCs) and/or mature T cells by neonates, followed by their migration to the thymus, as demonstrated in several studies [111,112,113,114].

Furthermore, the term “cocooning” strategy means vaccinating for pertussis to anyone who comes in close contact with an infant [99]. Recent data demonstrate that the most significant impact in preventing severe pertussis in neonates comes from vaccinating mothers, followed by fathers, with grandparents playing a smaller role. Siblings’ importance varies, and the decreasing immunity in vaccinated children requires further investigation. Additionally, non-household sources of pertussis have been well-documented, emphasizing the potential limitations of the cocoon strategy in preventing severe disease in infants [46]. Cocooning is difficult, frequently ineffective and it has considerable logistical obstacles, it can be too expensive to make sure that everyone who is around an infant is vaccinated.

Despite the high pertussis vaccination coverage in developed countries, such as the United States, Canada, and Australia, and a low rate of pertussis incidence for many years [115], the incidence of pertussis has been on the rise again, with outbreaks in some places, which is referred to as “re-emergence of pertussis”.

Pertussis remains endemic worldwide, even in regions with high vaccination coverage. It has reemerged in several countries with effective vaccination programs, particularly those that have switched from whole-cell pertussis vaccines to acellular pertussis formulations. This includes countries like the United States, Austria, Croatia, Norway, and Denmark [7,116,117]. While there is limited data on the global burden of pertussis, it is generally believed that the highest disease rates occur among young children in countries with low vaccination coverage, mainly in developing nations. In industrialized countries, the reported incidence of pertussis is highest among infants who are too young to receive the vaccine.

## 5. Recent Epidemiology

**Figure 2:**
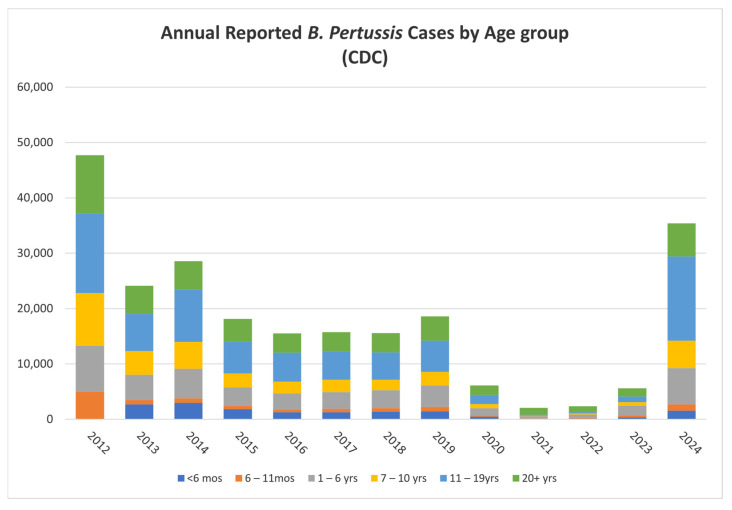
Annual *B. pertussis* cases by age group (CDC).

Pertussis remains an endemic disease worldwide with cyclical peaks of disease spread every three to five years. This is further supported by annual reported *B. pertussis* cases by the CDC (https://www.cdc.gov/pertussis/php/surveillance/index.html (accessed on 3 February 2025)) presented in Figure 2.

According to the European Centre for Disease Prevention and Control (ECDC), in 2022 there were more than 2623 cases of pertussis in Europe, with one death [118,119,120,121,122].

**Figure 3:**
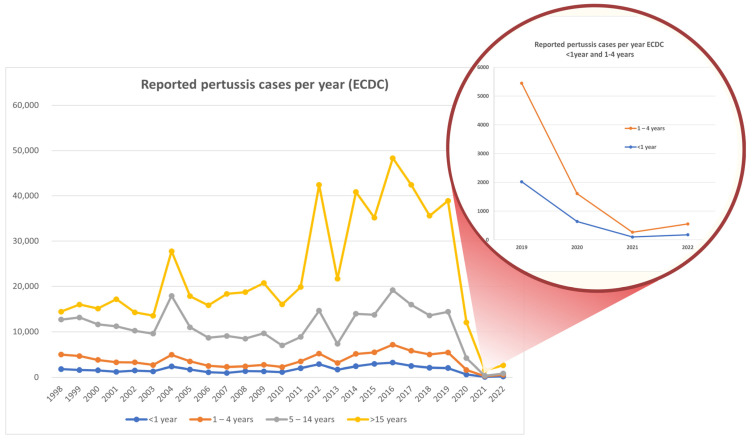
Total pertussis cases per year (1998–2022) in European pediatric population per age group (<1 year, 1–4 years, 5–14 years, >15 years) according to European Centre for Disease Prevention and Control (ECDC) (https://atlas.ecdc.europa.eu/public/index.aspx (accessed on 20 December 2024)).

**Figure 4:**
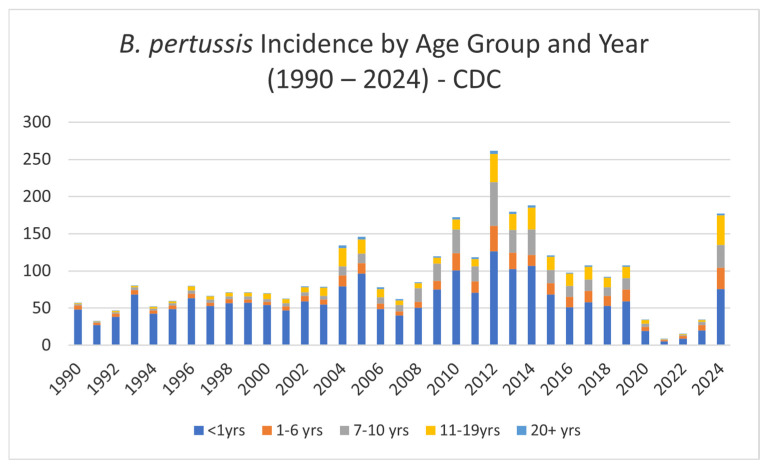
Annual *B. pertussis* incidence by age group and year (https://www.cdc.gov/pertussis/php/surveillance/index.html (accessed on 3 February 2025)).

Total pertussis cases per year from 1998 to 2022 in the European pediatric population per age group (<1 year, 1–4 years, 5–14 years, >15 years) according to the European Centre for Disease Prevention and Control (ECDC) are presented in Figure 3 and highlight a notable, surprising robust increase in pertussis cases in adolescents from 2012 until the beginning of COVID-19 pandemic in late 2019. Additionally, the annual *B. pertussis* incidence by age group, according to CDC data, is presented in Figure 4.

After a few years of restricted distribution in the EU/EEA—especially during the COVID-19 pandemic—in 2023 and in 2024 an outbreak has emerged of over 25,000 cases of pertussis in 2023, and over 32,000 cases between January and March 2024 [122]. Comparable figures were noted in the years 2016 (41,026) and 2019 (34,468).

**Figure 5:**
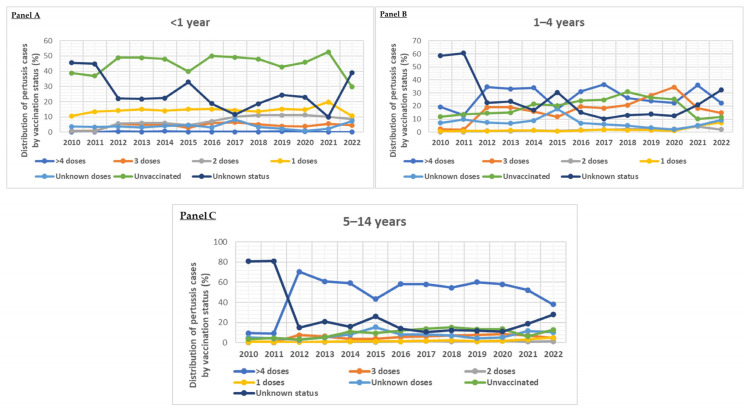
Distribution of pertussis cases per year (2010–2022) in European pediatric population according to vaccination status per age group (<1 year: (**Panel A**), 1–4 years: (**Panel B**), >4–14 years: (**Panel C**)) according to European Centre for Disease Prevention and Control (ECDC) (https://atlas.ecdc.europa.eu/public/index.aspx (accessed on 20 December 2024)).

In the past, the EU/EEA countries have recorded the greatest prevalence of pertussis in newborns (less than 1 year old). A rise in infants has been noted in 2023–2024, accompanied by significant increases in those aged 10–14 and 15–19, and, to a lesser extent, with rises in those aged 5–9 and 1–4 [122]. The incidence has stayed comparatively low among adults (over 20). There are some differences in the age distribution by nation, which could be caused by various booster dose schedules, differing pertussis vaccination regimens, varying levels of laboratory confirmation implementation in various age groups, and differing age groups targeted for surveillance. The distribution of pertussis cases per year (2010–2022) in the European pediatric population according to vaccination status per age group is presented in Figure 5.

In 17 countries (Austria, Belgium, Bulgaria, Cyprus, Estonia, Germany, Greece, Hungary, Ireland, Italy, Lithuania, Malta, the Netherlands, Portugal, Romania, Sweden, and Slovakia) in 2023–2024, infants made up the group with the highest incidence. The age groups with the second and third largest prevalence across these nations ranged widely, ranging from children 1–4 years old to adolescents (15–19 years old), 5–9 years old, and 10–14 years old.

Three countries—Croatia, Denmark, and Luxembourg—saw the highest occurrence in children aged 10 to 14 years, followed by adolescents aged 15 to 19 years. Two other countries—Czechia and Slovenia—saw the highest incidence in adolescents aged 15 to 19 years, followed by children aged 10 to 14 years. In Spain, the highest frequency was seen in infants in 2023, but the highest incidence was seen in children ages 1–4 in early 2024. In Norway, the highest occurrence occurred in 2023 among teenagers aged 15 to 19 and in 2024 among children aged 10 to 14.

In Greece according to the National Public Health Organisation’s new data (published on 6 June 2024) from the beginning of the year 2024 and until 30 May 2024, 230 cases of pertussis have been reported to EODY, while in the year 2023, only 9 cases were reported. It is worth noting that 133/230 (57.8%) concern children and adolescents <18 years of age, while 58/230 (25.2%) concern infants <12 months of age. Finally, 34/230 (14.8%) involve infants <2 months of age, 2 of which ended up while at least 3 required hospitalization in the neonatal intensive care unit (NICU) and intensive care unit (ICU) [123]

In the vaccine era (from the 1940s to the present), pertussis epidemics have occurred in 3- to 4-year cycles even in areas with high vaccination coverage, likely due to the periodic fluctuation of population immunity. This cyclical pattern may be influenced by waning immunity over time, as well as changes in vaccine coverage and the emergence of vaccine-resistant strains. Infants are at greatest risk of severe disease and death and almost all deaths in European countries have been recorded in infants under three months of age [42,120,124,125].

Between 2011 and 2022, pertussis-related mortality in Europe totaled 103 reported deaths, with the majority occurring in the most vulnerable age groups—69 (67%) in infants under 6 months and 25 (24%) in adults aged 60 years and older. The distribution of deaths fluctuated over the years, with peak infant mortality rates observed in 2012 and 2016 in accordance with the spikes in *B. pertussis* cases in these years, respectively, according to ECDC data. More recently, between January 2023 and April 2024, an additional 19 deaths were documented, including 11 (58%) in infants and 8 (42%) in older adults (≥60 years). This continued mortality burden, combined with a sharp increase in pertussis incidence across all age groups, underscores ongoing gaps in immunization coverage, particularly among pregnant women and adults. These trends highlight the urgent need for reinforced vaccination strategies to protect the most at-risk populations and curb the resurgence of pertussis in Europe [43].

Using the electronic WHO/UNICEF Joint Reporting Form (e-JRF) [52], the WHO tracks the coverage of pertussis vaccinations in the EU/EEA. Every year, information is gathered regarding the third and fourth doses of vaccinations containing pertussis. The third dosage of the diphtheria–tetanus toxoid and pertussis-containing vaccine (DTP3) is still well vaccinated against in EU/EEA nations, according to the most recent data (2022). This indicates a good level of protection for children in nations (Belgium, Bulgaria, Croatia, Cyprus, Estonia, Greece, Hungary, Lithuania, Malta, Poland, Latvia, and Portugal) that followed the 3p primary vaccine schedule at six months of age, or for infants in nations (Austria, Czechia, Denmark, Finland, France, Germany, Iceland, Luxemburg, Netherlands, Norway, Slovakia, Slovenia, Spain, Sweden, and Italy) that followed the 2*p* + 1 primary immunization schedule at eleven or twelve months of age [118]. The computed median value at the EU/EEA level is currently 94% coverage, with a trend that has been found to decrease from 97% in 2012 to 94% in 2022.

However, data about vaccination coverage for the fourth dose of the DTP4 vaccine, which contains a pertussis and tetanus toxoid, are extremely rare [27]. Depending on whether a 3*p* or 2*p* + 1 main schedule is chosen, this indication shows the vaccination coverage for the primary booster given at the age of 24 months or during preschool or primary school.

Despite the fact that all EU/EEA countries, except for Bulgaria, Estonia, Finland, Malta, and Slovakia, have adopted the recommendation, only nine countries provided the maternal immunization coverage data via the ad hoc data call through EpiPulse for this risk assessment.t. Reported coverage rates for 2023 varied between 1.6% and 88.5%, based on estimates from different years. Furthermore, there are no available data on uptake rates among adolescents and adults. Regarding uptake rates in the adolescent and adult age ranges, there are no data available [43].

Interpreting the data in Europe and around the world, pertussis is endemic; outbreaks are reported every three to five years, with summertime being the peak season. The low incidence of pertussis in the EU/EEA between 2020 and 2022 [5,27] may have contributed to the present spike in cases by making a larger percentage of the population susceptible to the illness.

In fact, low-level endemic, interseasonal transmission that functions as a natural booster may strengthen population immunity and reduce the likelihood of widespread outbreaks [57]. The childhood vaccination programs have very high vaccination rates, yet there has been a slight overall drop in the use of the third dose of pertussis-containing vaccinations. There could be sub-national variances as well as differences in how a life course strategy for pertussis immunization is implemented. The coverage of maternal vaccinations is moderate–low, according to preliminary statistics [122].

Many non-pharmaceutical interventions (NPIs), implemented to limit the spread of COVID-19 have proven to be highly effective in reducing case numbers, particularly when introduced early in the pandemic. These measures include travel bans and restrictions, school and workplace closures, isolation of infected individuals, quarantine of exposed individuals, social distancing, and the cancellation of mass gatherings. The timely and coordinated implementation of these interventions has been instrumental in reducing viral transmission, significantly contributing to pandemic control and alleviating the burden on healthcare systems. During the COVID-19 pandemic, these mitigation strategies were also associated with significant downward trends in pertussis disease indicators at both global and regional levels [126,127].

According to GBD 2021, the global age-standardized incidence rate (ASIR) of pertussis showed a decline of −1.75% (95% CI: −1.85% to −1.65%) per year between 1990 and 2019. However, from 2019 to 2021, ASIR experienced a significantly steeper reduction of −39.73% (95% CI: −46.95% to −31.53%), indicating a decline rate approximately 20 times faster following the onset of the COVID-19 pandemic [127]. At the same time, several infectious diseases, particularly respiratory infections, saw a sharp decline in incidence following the implementation of NPIs, primarily due to the reduction in pathogen transmission resulting from social distancing.

However, while NPI measures positively impacted the reduction of infectious disease transmission, research suggests they may have also increased the population’s susceptibility to certain infections. Reduced natural exposure to pathogens may have contributed to waning immunity against diseases unrelated to SARS-CoV-2, leading to subsequent increases in cases and shifts in the seasonality of diseases such as respiratory syncytial virus (RSV), influenza, norovirus, pertussis, and pneumococcal disease [76,128,129]. Low rates of norovirus infections were recorded in the UK during lockdown periods, which may have contributed to increased population susceptibility [130]. Following the lifting of restrictive measures, a rapid increase in the incidence of certain infections was observed, highlighting the potential long-term effects of NPIs on the epidemiology of infectious diseases [131]. Furthermore, during the COVID-19 pandemic, mitigation measures such as lockdowns and school closures led to a significant reduction in the uptake of essential childhood vaccinations, including MMR, diphtheria–tetanus–pertussis (DTP), and HPV vaccines. The impact was especially severe in countries with the strictest containment measures. Global data revealed that in 2020, DTP3 vaccine coverage dropped to 83%, leaving 22.7 million children unprotected [57].

Following the decreased circulation of the disease during the COVID-19 pandemic, suboptimal vaccination coverage during this period, and waning immunity, a resurgence of pertussis cases has been reported worldwide [7,43,116,117].

It is important to consider vaccination coverage and schedules (primary series and boosters) when interpreting the incidence of pertussis. The age distribution of patients may have changed as a result of prior adjustments to schedules and uptake over time. There is compelling evidence that protection against pertussis gained from vaccination and natural infection wanes with time [37,132]. This implies that people who have had vaccinations in the past may become infected [133]. For these reasons, caution needs to be exercised when comparing current (2023–2024) data with historical pertussis data.

## 6. Risk Assessment for the EU/EEA

This risk assessment was created using the most recent data available at the time of publication and adheres to the ECDC quick risk assessment methodology, which calculates the overall risk by combining the likelihood of infection with its consequences [3]. When a new health danger emerges, the likelihood of infection and the disease’s impact are evaluated, taking into account the features of the location (the country in which the illness is happening) and the individual (the prevalence of risk groups in the European population).

The likelihood of contracting pertussis and the possibility of contracting the disease later on are determined by a number of factors, such as the individual’s vaccination status (number of doses received and time elapsed since last dose), the vaccination status of close contacts, and the prevalence of *B. pertussis* in the specific community, environment, or age group. Even in the post-vaccine era, pertussis epidemics recur every three to five years, and the illness is currently regarded as endemic in the adult population. Pertussis has a basic reproduction number (R0) of 12–17 and it is considered to be a highly contagious disease [9]. A further risk factor could be limited access to financial resources. If a region or country is facing economic hardship or is a lower-income area within a wealthier nation, the inability to afford vaccines may elevate the risk of pertussis transmission. Financial constraints can reduce access to healthcare and immunization services, increasing the population’s susceptibility to vaccine-preventable diseases [134].

Unimmunized or partially immunized infants under six months of age are considered to be at high risk overall, with a high probability of exposure and a high impact. Infants typically contract an infection from an undiagnosed parent, elder sibling, or other caregiver [5]. In neonatal intensive care units, nosocomial epidemics can also happen. Infants six months of age or less are claimed to be involved in over 80% of hospitalizations. Furthermore, because of comorbidities like pneumonia, apnea, seizures, and encephalopathy, this age group has the greatest documented fatality rate. The case fatality rate of pertussis is 1–2% in newborns and infants up to two months of age [4]. A total of 95.5% of the deaths that the ECDC was notified of between 2011 and 2022 involved infants younger than six months of age. For this reason, we determine that there is a high impact of pertussis infection in this age range. According to the UK Health Security Agency (UKHSA), in recent years, confirmed pertussis cases in infants under three months, who are at the highest risk for severe disease and cannot be fully vaccinated, have shown a concerning trend. After peaking at 407 cases during the 2012 outbreak (according to UKHSA, https://www.gov.uk/government/publications/pertussis-epidemiology-in-england-2024/confirmed-cases-of-pertussis-in-england-by-month (accessed on 22 December 2024)), the introduction of maternal vaccination initially reduced cases. However, reported cases rose from 2 in 2022 to 48 in 2023, although still lower than the 83 cases recorded in 2019 [32,135,136]. The highest incidence continues to be among this age group, with 379 confirmed cases from January to July 2024, compared to 237 cases during the same period in 2012. Since maternal vaccination began in 2012, there have been 30 deaths in infants under one year from confirmed pertussis, including 9 fatalities in early 2024, predominantly among those whose mothers were unvaccinated during pregnancy [135].

Maternal vaccination during the second and third trimester of pregnancy, currently recommended in 9 European countries (Belgium, Czechia, Denmark, Germany, Ireland, Portugal, Romania, Slovenia, and Spain), has high effectiveness in preventing pertussis in the first two months of life. The majority of European nations currently advise maternal immunization during the second and third trimester of pregnancy since it is highly effective in avoiding pertussis in the first two months of life [98,101,107,136]. Strategies such as “cocooning”, in which immunizing a newborn’s caregivers are advised, are very logistically complex and frequently ineffective [137,138].

The overall risk is deemed to be moderate for children under the age of fifteen and for infants older than six months if they have received only a partial or no vaccination. According to ECDC data, this age group accounts for 43% of the currently reported cases of pertussis in 2023 and the first few months of 2024. This suggests that this age group may have a partial immunization history and some decreasing immunity following the primary schedule. However, children who have recently finished the recommended immunization series with a pertussis-containing vaccine (often DTaP) are also likely to be included in this group, as they have a high level of immunity against infection. For these kids, there will not be much risk overall. In general, pertussis has little effect on this age range [43].

Finally, those who are 65 years of age or older, people of any age who have underlying medical disorders including asthma, chronic obstructive pulmonary disease (COPD), or people who are immunosuppressed may have a more severe case of pertussis and a higher hospitalization rate, most often from pneumonia. Older individuals (60 years of age or older) accounted for 42% (8/19) of the deaths reported to the ECDC in 2023 and thus far in 2024. This category’s total risk is rated as moderate due to the moderate probability and impact [139,140].

## 7. Recommendations

Recognizing the significance of attaining high vaccination coverage among pregnant women, it is critical to identify and address the variables affecting adherence to immunization programs.

1. The ECDC recommends that standard prenatal visits include vaccine counseling for expectant mothers. Since a child’s health is a key factor in a mother’s decision to accept vaccination, counseling should primarily focus on its benefits. Pregnant women rely on healthcare professionals for clinical guidance and to ensure the well-being of their offspring. Healthcare providers recognize that their recommendations play a vital role in promoting vaccination adherence during pregnancy. Therefore, doctors should ensure that patients fully understand the information provided and remain available to address any further questions. Maternal vaccination offers a safe and effective means of providing passive immunoprotection to newborns. However, immunization rates among pregnant women remain inconsistent and suboptimal.
2. According to ECDC’s suggestions, vaccination adherence can be improved by having educational materials like posters or pamphlets in hospital waiting areas, making vaccinations easily accessible (by giving them out during routine visits, for example), and providing ongoing training to medical staff to increase their confidence and knowledge.
3. One of the main goals of immunization programs is to protect newborns from pertussis, which can cause fatal complications and significant sickness. One of the most important ways to avoid pertussis is to vaccinate children and adults as early as the second month of life, making sure they receive all the doses recommended by the National Vaccination Programme (NVP).
4. The National Vaccination Committee (NCC) and the National Public Health Organization (NPHO) advise: Medical professionals—including pediatricians, neonatologists, obstetricians-gynecologists, general practitioners, pathologists, and pulmonologists—must remain vigilant in promptly initiating appropriate macrolide therapy when clinical suspicion is high. This is especially crucial for adults experiencing a persistent paroxysmal cough, even in the absence of other symptoms. It is important to note that delaying antibiotic treatment renders it ineffective. Additionally, all high-risk individuals who have been in contact with a confirmed pertussis case should receive prophylactic antimicrobial therapy, regardless of their immunization history or previous illness.
5. According to the American College of Obstetricians and Gynecologists and relevant scientific societies, the Tdap vaccine should be administered to all pregnant women between 27 and 36 weeks of gestation. Additionally, unvaccinated women should receive postnatal vaccination before discharge from the maternity hospital, regardless of the time elapsed since their previous Td/Tdap vaccination. This is the only preventive measure for young infants under 3 months of age, who are at the highest risk for morbidity and mortality due to pertussis [139].
6. Prompt vaccination of all family members who come into touch with newborns/infants and who are not completely vaccinated against pertussis, at least two weeks prior to the interaction.

## 8. Conclusions

In conclusion, pertussis (whooping cough) is a significant cause of morbidity and mortality, particularly affecting infants under three months of age, who are too young to be vaccinated.

Protecting infants, particularly neonates and those under three months of age, is of paramount importance due to their heightened vulnerability to infectious diseases. At this stage, infants are often too young to have initiated or completed their vaccination schedules, resulting in limited immunity. This population is at significant risk for severe complications from preventable diseases such as pertussis, influenza, and measles. Early protection for this group reduces morbidity and mortality from vaccine-preventable diseases.

A comprehensive approach is necessary to effectively address the resurgence of pertussis, which is a leading cause of illness, hospitalization, and death in infants. Key strategies include vaccinating pregnant women to enhance newborn immune protection, ensuring infants receive timely vaccinations, providing antibiotic post-exposure prophylaxis to close contacts, and launching targeted vaccination campaigns for those who have missed booster doses. Additionally, promoting active surveillance and screening of symptomatic individuals is critical for minimizing exposure and controlling disease spread. The rising rates of infant hospital admissions and mortality underscore the urgent need to prioritize these interventions within public health initiatives.

Monitoring the impact of maternal pertussis vaccination recommendations on the epidemiology of pertussis in infants under four months of age will be essential. Improved monitoring systems that document vaccination history, pertussis strains, and outbreak data can provide a more complete picture of pertussis epidemiology. This knowledge will enable healthcare providers, including obstetricians and pediatricians, to adapt their practices and offer the best available care to their patients based on current data. There is also scope to strengthen monitoring and surveillance systems at international, national, and sub-national levels to ensure the availability of up-to-date data. This will guide policy and planning at regional and national levels, optimizing vaccine coverage and impact while identifying areas with concentrated low coverage. Additionally, addressing challenges such as vaccine availability, cost barriers, and vaccine hesitancy, and implementing strategies to overcome these obstacles is essential for improving vaccination outcomes.

## Figures and Tables

**Table 1:** Innovative vaccine strategies and delivery methods.

Category	Description
**Vaccine Platforms**	
Live Attenuated Vaccines	Effective in disease control but pose safety concerns, especially for immunocompromised individuals.
Whole-Cell Pertussis (wP) Modifications	Focused on reducing side effects while maintaining strong immune responses through refined bacterial inactivation and LPS modifications [65,66].
Acellular Vaccine Enhancements	Requires careful selection of antigens and adjuvants to boost immune responses. Novel antigens like BrkA and BcfA show promise [67,68].
Adjuvant Innovations	Shift towards Th1/Th17-inducing adjuvants (e.g., β-glucan, CpG 1018, AS01, and new saponin-based adjuvants) to enhance cellular immunity. One of the major drawbacks of adjuvants is their potential to cause adverse reactions in humans. A novel saponin-based adjuvant, called TQL1055 completed Phase I trials in humans, showing a safety profile similar to the current aP vaccine, indicating a promising alternative [69].
**Novel Vaccines in Development**	
BPZE1 (Live Attenuated)	In Phase 2b trials, demonstrating strong mucosal and systemic immune responses [70].
Bbvac (Live Attenuated)	Designed for long-term protection against multiple Bordetella species [70].
Outer membrane vesicles (OMV)-Based Vaccines	Contain a broad antigen profile that triggers Th1, Th2, and Th17 responses, providing protection against circulating Bordetella strains [71].
Bacterial Ghosts	Empty bacterial envelopes showing protective effects in animal models, though more research is needed [72].
**Routes of Administration**	
Intranasal	An attractive approach for eliciting strong mucosal immune responses. (inducing local IgA and IL-17 responses, though regulatory challenges remain) [70].
Oral	Convenient for administration and capable of triggering local immune responses, but stability and tolerance issues need to be addressed [73].
Epicutaneous (Skin Patch)	Utilizes Langerhans cells for immune activation; early trials indicate strong immune responses and good safety profiles (phase I clinical trial) [63].
**Future Considerations**	To maximize effectiveness, future vaccines should be tailored to specific at-risk populations, including immunocompromised individuals and pregnant women, by optimizing formulation, delivery methods, and dosing schedules.

## Data Availability

Data are contained within the article.
